# Evolution of *Salmonella* within Hosts

**DOI:** 10.1016/j.tim.2018.06.001

**Published:** 2018-12

**Authors:** Jennifer R. Tanner, Robert A. Kingsley

**Affiliations:** 1 Quadram Institute Bioscience, Norwich Research Park, Colney, Norwich, UK

**Keywords:** *Salmonella*, evolution, pathogenesis, adaptation

## Abstract

Within-host evolution has resulted in thousands of variants of *Salmonella* that exhibit remarkable diversity in host range and disease outcome, from broad host range to exquisite host restriction, causing gastroenteritis to disseminated disease such as typhoid fever. Within-host evolution is a continuing process driven by genomic variation that occurs during each infection, potentiating adaptation to a new niche resulting from changes in animal husbandry, the use of antimicrobials, and emergence of immune compromised populations. We discuss key advances in our understanding of the evolution of *Salmonella* within the host, inferred from (i) the process of host adaptation of *Salmonella* pathovars in the past, and (ii) direct observation of the generation of variation and selection of beneficial traits during single infections.

## Highlights

*Salmonella* is a bacterial pathogen with remarkable diversity in its host range and pathogenicity due to past within-host evolution in vertebrate species that modified ancestral mechanisms of pathogenesis.

Variation arising during infection includes point mutations, new genes acquired through horizontal gene transfer (HGT), deletions, and genomic rearrangements.

Beneficial mutations increase in frequency within the host and, if they retain the ability to be transmitted to subsequent hosts, may become fixed in the population.

Whole-genome sequencing of sequential isolates from clinical infections reveals within-host HGT and point mutations that impact therapy and clinical management.

HGT is the primary mechanism for evolution in prokaryotes and is synergised by complex networks of transfer involving the microbiome.

Within-host evolution of *Salmonella*, resulting in new pathovars, can proceed in the absence of HGT.

## The Significance of Within-Host Evolution

**Figure 1:**
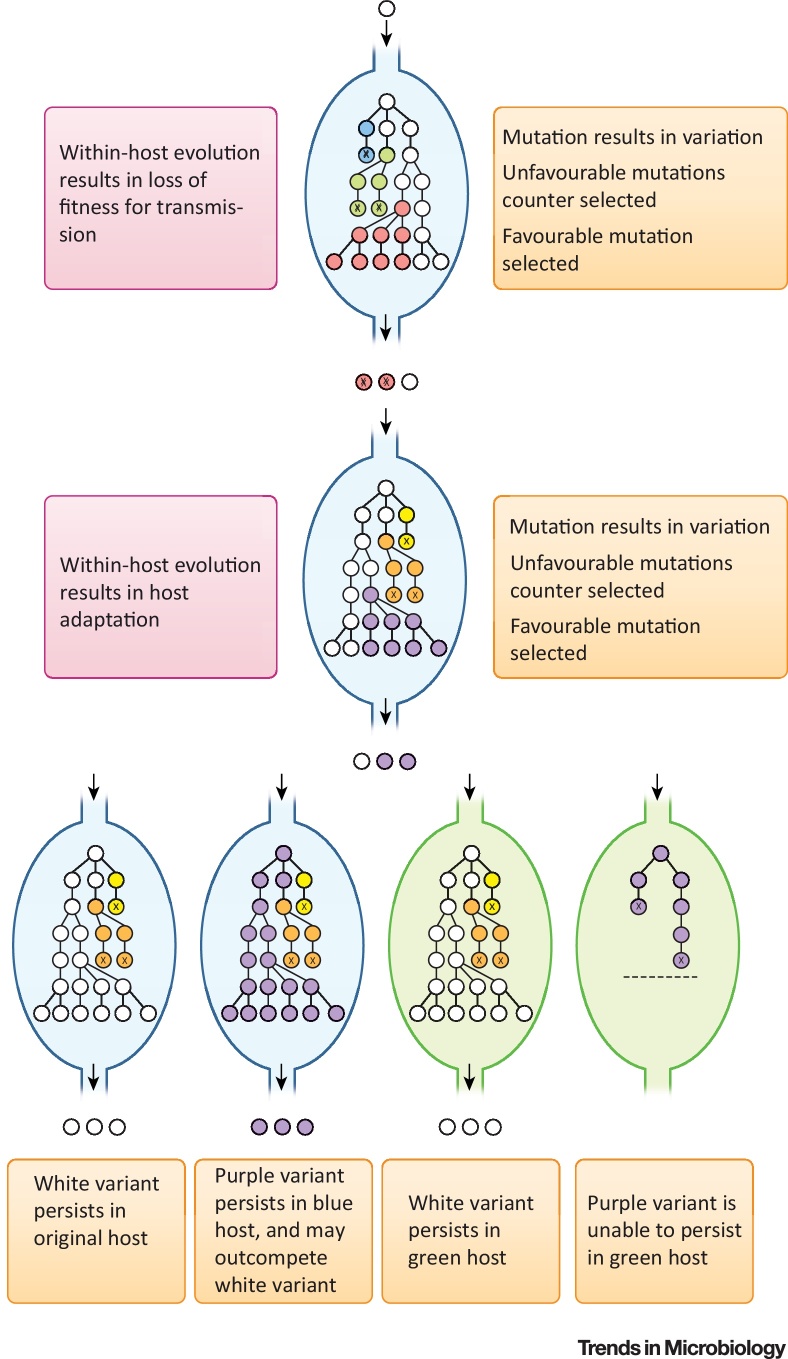
Within-Host Evolution of *Salmonella* and Its Consequences. Illustration of *Salmonella* (white circle) entering a host animal (blue or green outline) resulting in the emergence of variation during replication (colour-filled circles). Variants with beneficial traits increase in frequency due to diversifying selection (red and purple circles), while those with detrimental or neutral traits are lost from the population by genetic drift or purifying selection (marked ‘X’). However, within-host evolution, resulting in the increased frequency of beneficial mutations, may not be beneficial outside of the host and counter-selected due to its loss in fitness for transmission (red circle with ‘X’) or result in increased fitness (purple circle) in one host (blue outline) but decreased fitness (purple circle marked with ‘X’) in a second host species (green outline), in which case host restriction may occur.

During infection in its many mammalian, avian, amphibian, and reptile hosts, *Salmonella* is in constant competition with other microorganisms for a niche that provides nutrients for replication, and must contend with the host immune defences that limit or prevent spread beyond the intestinal mucosa. *Salmonella* coevolved with animal hosts for millions of years since diverging from a common ancestor with *Escherichia coli* (Box 1), and it is likely that most mutations that arise during infection are either neutral or detrimental, and therefore either fixed at low frequency by **genetic drift** (see Glossary) or rapidly lost (Figure 1). This is illustrated by the scarcity of nonsynonymous compared to synonymous substitutions (dN/dS <1) during short-term evolution of *Salmonella* [1], signifying that nonsynonymous single-nucleotide polymorphisms (SNPs) are subject to **purifying selection**. However, evolution of *Salmonella* may occur when a mutation provides an advantage in a novel niche in which to replicate, and if this also provides an opportunity to transmit to a subsequent host, it may be a stable event. Novel niches take many forms, including nutrient availability, the presence of antimicrobial, alternative tissue or organ sites in the same host, such as with disseminated infections, alternative hosts with altered immune status, or an alternative host species. Regardless of its nature, entry into a new niche alters natural selection on sequence variation arising from replication errors and horizontal gene transfer (HGT), resulting in an increase in the frequency of beneficial mutations, termed diversifying selection (Box 2). Observations of within-host evolution of *Salmonella* that led to the emergence of distinct pathovars and **disseminated disease**, indicate that this is normally associated with a decrease in host range, because mutations benefitting replication in systemic sites in one host species do not result in the same benefit in other host species. This creates an apparent paradox whereby a decrease in the population size of susceptible hosts accompanies host adaptation, a seemingly evolutionary unstable event. In practice, there is a trade-off for the relative benefit to transmission in a single host species population, and the decrease in host range due to loss of fitness in other hosts [2]. In the case of *Salmonella enterica* **serovar** Typhi (*S*. Typhi), host restriction to humans was accompanied by a distinct transmission strategy involving dissemination to systemic sites to gain access to the gall bladder, establishing long-term persistence. The decrease in number of susceptible hosts due to host restriction was counterbalanced by the increased longevity of transmission.

Box 1 **Evolution of Salmonella Pathogenesis**

The genus *Salmonella* comprises over 2500 serovars in two species, *S. bongori* and *S. enterica*; the latter contains subspecies I, II, IIIa, IIIb, IV, VI, and VII. All *Salmonella* serovars cause disease essentially by the same mechanisms, because soon after divergence from a common ancestor, *Salmonella* acquired key virulence genes. These include two type III secretion systems (T3SS-1 and T3SS-2) encoded on *Salmonella* pathogenicity islands 1 and 2 (SPI-1 and SPI-2) that enable invasion of host epithelium and subsequent intracellular survival 88, 89. T3SSs are supramolecular structures, resembling a syringe, that translocate effector proteins across host cell membranes. Initially, *Salmonella* coevolved with cold-blooded host species, with subspecies I in particular expanding its host range to include warm-blooded animals, the major zoonotic reservoir for human infection. Serovars of subspecies I consequently account for the vast majority of human infections. Evolution of subspecies I within these hosts included the acquisition of around 216 genes [90], including *shdA* involved in persistent intestinal colonisation [56]. *Salmonella* primarily evolved as a gastrointestinal pathogen, with infection limited to the intestinal lumen, intestinal mucosa, and associated lymphoid tissue. Invasion of the intestinal mucosa is a strategy to generate an intestinal niche in the lumen that favours the replication of *Salmonella* over the intestinal microbiota. Invasion elicits a host inflammatory response [5], exploiting host-derived metabolites and resisting antimicrobial defences specific to the inflamed intestine [91]. Gastroenteritis is acute and self-limiting; nonetheless, the short-term proliferation of *Salmonella* in the intestine ensures transmission. However, in some *Salmonella* serovars, within-host evolution has resulted in adaptation to an alternative transmission lifestyle that does not rely on replication in the intestine, but rather, dissemination to systemic sites, niches devoid of competing microbiota allowing persistence that can last the lifetime of the host. Within-host evolution on multiple occasions resulted in a number of host-adapted serovars (pathovars) often with apparent convergence in pathogenesis, in the same or distinct host species, and reflected in the convergence of their genome sequence 92, 93, 94. For example, *S*. Typhi and *S*. Paratyphi A cause typhoid and paratyphoid, similar diseases restricted to humans; *S*. Gallinarum is associated with a disease called fowl typhoid; *S*. Abortus-ovis is associated with abortion in sheep, due to a tropism for the placenta; and *S*. Dublin and *S*. Choleraesuis are associated with bacteraemia in cattle and pigs, respectively. In each case, within-host evolution has selected for variants that evade detection by the host innate immune system, with concomitant blunting of the inflammatory response in the intestinal mucosa that facilitates dissemination to systemic sites.

Alt-text: Box 1

Box 2 **Sources of Variation and Selection of Traits within the Host**

Genome sequence variation results from point mutations, horizontal gene transfer (HGT), deletions, duplications and rearrangements. Point mutations arise at a rate of around 1 × 10^−9^ to 1 × 10^−10^ per base pair per generation [95], and although extremely low, the large overall population size during infection, and the short generation time, provides the opportunity for diversity to arise during a single infection. Although the HGT rate is comparable to point mutation, HGT dominates prokaryotic evolution because the impact on heritable traits is greater due to the introduction of new functional genes 96, 97. The dominance of HGT on *Salmonella* evolution is apparent from the observation that *S*. Typhi and *S*. Typhimurium share an average nucleotide identity of around 99%, yet around 15% of their genes are serovar-specific 13, 14. Genes affected include prophage, pathogenicity islands, ICEs, transposons, IS elements, and plasmids [90]. Despite the clear importance of HGT in the evolution of *Salmonella*, especially over longer time scales since serovars shared a common ancestor, the emergence of new pathovars over short time scales can occur with little or no HGT [38]. In these cases, point mutations in a genome sequence appear more important, although their impact on function is difficult to predict. Insight into the functional divergence of proteins using a profile-based method with hidden Markov models is a promising approach [98]. Mutations in transcriptional regulators and regulatory sequences have the potential to have a broad ranging impact on traits of the organism. Indeed, pathovars of *Salmonella* have diverse regulatory networks, and are also likely to play an important role in the evolution of *Salmonella* [32]. For example, the PhoP regulon, which includes ∼3% of the *S*. Typhimurium genome, is known to be highly plastic, with transcriptional rewiring affecting gain and loss of interactions with shared sets of genes in members of the Enterobacteriaceae [99]. Regardless of the mechanism, variation that is unfavourable is rapidly lost from the population by purifying selection, while beneficial mutations increase in frequency by the process of **diversifying selection**. Neutral mutations are normally fixed in the population at low frequency, because of the large effective population size of bacterial species. However, during infection, the population goes through bottlenecks, potentially resulting in increased frequency of neutral mutations 100, 101.

Alt-text: Box 2

Within-host evolution is also constrained by the need for *Salmonella* to survive in the environment and to be transmitted to subsequent hosts. Consequently, if a mutation results in greater replication within the host, but confers a fitness cost in the environment or for colonisation of a subsequent host, the variant will not be viable (Box 2). An elegant example are variants with mutations leading to the loss of expression of energetically expensive virulence genes such as the type III secretion system-1 (T3SS-1, Box 1), required for invasion of host cells. These variants replicate more rapidly than those expressing T3SS-1 *in vitro* and *in vivo* [3]. Invasion of the intestinal mucosa by the *Salmonella* population expressing the T3SS-1 is an example of self-destructive cooperation [4], because bacteria that invade and elicit inflammation do not benefit from the resulting inflamed intestinal niche 5, 6, 7. This is open to exploitation by faster replicating variants that do not express T3SS-1, and indeed irreversible loss of expression of T3SS-1 is observed at a surprisingly high frequency during infection due to mutation of HilD, the master-regulator of T3SS-1 [3]. The evolutionary instability of invasion appears to be counteracted by phase variable expression of the T3SS-1. That is, only a proportion of *Salmonella enterica* serovar Typhimurium (*S*. Typhimurium) in the population express the T3SS-1, the remainder compete with the *hilD* mutant variants in the lumen, yet are capable of switching back to the T3SS-1 phase ON mode in subsequent hosts [3]. While **phase variation** of other supramolecular structures such long polar fimbriae (LPF) has been proposed to contribute to maintaining the ability to infect a broad host range and evade cross immunity [8], phase variation of LPF that contribute to colonisation of lymphoid tissue of the intestinal mucosa [9] may also counter self-destructive cooperation. Phase variation of *lpf* varies depending on culture conditions, with the phase ON to OFF frequency dominant during replication in the intestine, providing a phase OFF population in the lumen that can compete with *lpf* mutants [10].

The emergence of variation and within-host evolution during infection is therefore a double-edged sword. It provides a pool of both genotypic and phenotypic diversity capable of exploiting a new niche and averting extinction – for example, in the face of a sudden insult from antimicrobials by acquisition of resistance genes, but that is also capable of leading the pathogen down an evolutionary dead end in the pursuit of short-term advantage. Furthermore, evolution resulting in a sophisticated level of adaptation to a new host requires multiple independent adaptations that act in concert and are therefore unlikely to emerge during a single infection [11]. The source-sink model of pathogen evolution explains how this may occur from multiple rounds of selection as the pathogen cycles through occupation of source habitats, where the population is self-sustaining, and sink habitats where the population can only be maintained by continuous reintroduction from the source habitat [12]. The source-sink model can explain how multiple rounds of selection for beneficial mutations for colonisation of an alternative host may occur and eventually result in an exquisitely host-adapted pathogen that can then be self-sustained and genetically isolated from the ancestral pathogen variant.

## Within-Host Evolution Inferred from Host Adaptation of *Salmonella* Pathovars

The evolution of host-adapted variants of *Salmonella* from broad host range ancestors occurred on multiple occasions, typically by adaptation to a disseminated disease lifestyle. Comparative genomics of a broad host range serovar (e.g., *S.* Typhimurium) with a host-adapted serovar (e.g., *S*. Typhi) identified changes in genome sequence that occurred since divergence in pathogenic lifestyle 13, 14. This included point mutations impacting protein sequence, promoters, and regulatory sequences, genome rearrangement, deletions, and insertions.

### The *S*. Typhi Paradigm

**Figure 2:**
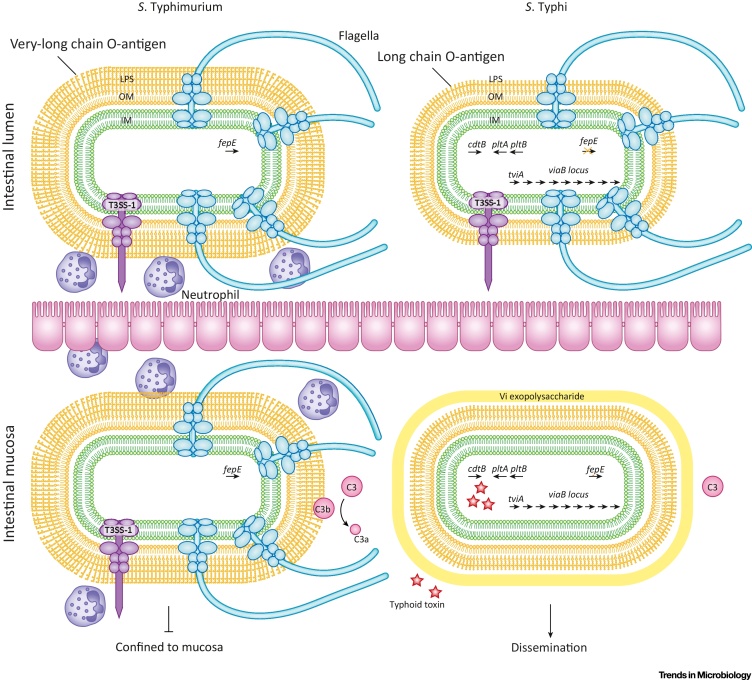
Within-Host Evolution Inferred from *Salmonella* Typhi Host Adaptation. Broad host range serovar *S*. Typhimurium and host-restricted serovar *S*. Typhi initiate infection by invasion of the intestinal mucosa mediated by T3SS-1-dependent invasion of the epithelium. The expression of flagella and lipopolysaccharide (LPS) with very long O-antigen chains by *S*. Typhimurium and the concomitant transfer of effector proteins by the T3SS-1, induces an acute host inflammatory response that prevents dissemination of *S*. Typhimurium to extraintestinal sites. Key to limiting S. Typhimurium dissemination is the recruitment of neutrophils by both complement-dependent and -independent mechanisms – where complement activation occurs through the complement component 3 (C3) cleavage product, C3b, covalently interacting with the exposed O-antigen chains 17, 104. *S*. Typhi, on the other hand, evades detection by the host immune response to facilitate dissemination. Through the action of the acquired TviA regulator (encoded by *tviA*), *S*. Typhi downregulates the expression of flagella and T3SS-1 while activating the expression of genes (black arrows) encoding Vi exopolysaccharide (*viaB* locus) 105, 106, 107. To potentially accommodate the Vi capsule, S. Typhi no longer produces LPS with very long O-antigen chains, due to disruption (orange ‘X’) of *fepE* encoding a regulator of O-antigen chain lengths. These changes in gene expression and coding capacity work together to reduce the host immune response by inhibiting complement activation (C3) and reducing proinflammatory cytokine production. For pathogenesis in the human host, *S*. Typhi produces the typhoid toxin (red stars) upon internalization via expression of the acquired and subsequently adapted *cdtB*, *pltA*, and *pltB* genes.

*S*. Typhi has 601 genes for which there are no orthologues in *S*. Typhimurium [14]. The acquisition of two clusters of genes in particular had a profound impact on the evolution of *S*. Typhi: a large integrative conjugative element (ICE) called *Salmonella* pathogenicity island 7 (SPI-7) [15], and three genes encoding a novel A_2_B_5_ toxin (Figure 2) [16]. SPI-7 encodes a number of genes, in the *viaB* locus, that mediate evasion of detection by the host innate immune system by either concealing or tightly regulating the expression of conserved molecular patterns of bacteria recognised by the immune system. The *viaB* locus encodes the Vi exopolysaccharide capsule that prevents complement activation by inhibiting binding of natural IgM to cell-surface macromolecules 17, 18, 19. SPI-7 also encodes a regulator, TviA, that not only controls biosynthesis of Vi [20] but also regulates expression of flagella and T3SS-1 [21]; after acquisition, TviA became integrated into the RcsB response regulatory pathway as an auxiliary protein. Downregulation of flagella and T3SS-1 expression after invasion of the intestinal mucosa by *S*. Typhi blunts the immune response in two ways. *S*. Typhi prevents detection of flagellin by TLR-5 that activates NF-κB signalling, and Naip5-Naip6/NlrC4/Caspase-1 inflammasome complex that activates cytokines interleukin 1β and interleukin-18-mediated inflammatory signalling pathways 21, 22, 23, thereby preventing detection of processes induced by T3SS-1 [24]. Artificial introduction of the *viaB* locus into *S*. Typhimurium results in decreased immune cell trafficking, decreased proinflammatory cytokines, and increased expression of the anti-inflammatory cytokines in experimental infections of mice, demonstrating the potential impact of a single HGT event to within-host evolution [25].

In a seemingly complex series of HGTs and subsequent mutations, *S*. Typhi acquired and evolved a novel A_2_B_5_ exotoxin 26, 27. The A subunit that exerts the toxic activity associated with the many symptoms of typhoid exhibits homology to CdtB, a cytolethal distending toxin present in the genomes of *Campylobacter* and some *E. coli* strains, suggesting that interactions in the intestine with other gut pathogens may have been the source of HGT [26]. The second A subunit, PltA, and the B subunit PltB, have extensive sequence homology with ArtA and ArtB, proteins from an A_1_B_5_ toxin present in a number of broad host range *Salmonella enterica* serovars, implicating a second acquisition during coinfection in the intestine. The unique A2 component of the toxin is made possible by an additional cysteine residue in the C terminus of PltA, which is not present in ArtA. The PltB subunit directs specific targeting of the toxin in the human host, as it binds sialylated glycans present in the human host, but lacks an additional glycan-binding site present in ArtB that broadens its binding to include glycans present in a wide range of mammalian hosts 28, 29. Specific targeting appears important to disease since a chimeric ArtB/PltA/CdtB toxin had reduced toxicity.

Deletions and point mutations also contributed to the evolution of *S*. Typhi by impacting genome degradation. Loss of function mutations may be either a result of reductive evolution, in which functions are no longer required for a new mode of pathogenesis, or adaptive evolution in which expression of a gene is detrimental, for example antivirulence genes [30]. *S*. Typhi lost nearly 5% (209 genes) of its original coding capacity during the process of adaptation to the human host and loss of virulence for alternative hosts [14]. Genome degradation impacted many functions, including genes involved in anaerobic metabolism 31, 32 required for outgrowth of *Salmonella* in the inflamed intestine during gastroenteritis [33]. Since inflammation in the intestine is not a common pathology in **typhoid fever**, the energy expense of expressing these genes led to reductive evolution. On the other hand, adaptive evolution resulted in disruption of the *fepE* gene that prevented expression of very long O-antigen chains of the cell envelope lipopolysaccharide that would otherwise interfere with deployment of the Vi polysaccharide capsule [34], and deletion of the ydiQRSTD operon that cooperates with Vi expression to moderate inflammation by preventing butyrate utilization [35].

### Recent Host Adaptation of *S*. Typhimurium Pathovariants

Emergence of host-adapted pathovariants of *S*. Typhimurium highlights that within-host evolution and adaptation to new hosts is an ongoing process. They share a common ancestor in the recent past, differ by around 500–1000 SNPs in the core genome, and exhibit limited gene flux outside of mobile genetic elements such as **phage**, ICEs, and plasmids 36, 37, 38. Therefore, in contrast to host-adapted serovars of *Salmonella*, pathovariants of *S*. Typhimurium evolved in the absence of acquisition of pathogenicity islands. For example, the **phage type DT**2 pathovariants, which are host restricted to pigeon and cause a typhoid-like disease, have an almost identical gene complement to broad host range variants of *S*. Typhimurium [38]. Perhaps the most significant adaptation of *S*. Typhimurium DT2 is a remarkable rewiring of its transcriptome that results in changes in gene expression in response to elevated temperature, typical of the avian host, including downregulation of flagella and motility genes, similar to that observed in *S*. Typhi 38, 39. Unlike in *S*. Typhi, where this is achieved by acquisition of the TviA regulator, no additional regulators are present in DT2, suggesting that convergent evolution is mediated by point mutations in pre-existing regulatory elements. A second example of the emergence of a pathovariant is *S*. Typhimurium ST313, associated with severe disseminated disease in sub-Saharan Africa [40]. Considerable genome degradation in these strains included genes involved in enteropathogenesis, reflecting the lack of intestinal involvement in these infections [41], multicellular behaviour implicated in environmental survival 42, 43, serum sensitivity [44], and dissemination [45]. Evolution of ST313 therefore involved selection for a variant that hyperdisseminates and has reduced enteropathogenesis 41, 46, 47.

## Direct Observations of *Salmonella* Within-Host Evolution

Catching evolution in action during natural infections is challenging as selection of variants with distinct heritable traits typically takes long periods of time, and traits often emerge by incremental steps that are difficult to measure. Nonetheless, within-host evolution can be observed when selection is strong, and results in a measurable phenotype such as drug resistance, or where chronic or recurring infections span relatively long periods, especially if this is accompanied by hypermutation [48]. Within-host evolution can also be observed in experimental infections, where strong selection pressure can be applied in a controlled manner and specific evolutionary events monitored [49].

### Within–Host Evolution in Clinical Infections

Although nontyphoid *Salmonella* infections are commonly acute and self-limiting, a meta-analysis of surveillance data in Israel found that around 2% of cases resulted in persistent infection, often associated with the inappropriate use of antibiotics [50]. Recurrence is considerably higher in disseminated disease caused by nontyphoid *Salmonella* (also called invasive NTS, iNTS disease) in HIV-infected adults, where recurrence is as high as 25% [51]. Whole-genome sequencing of pairs of strains from persistent clinical infections identified more sequence variation than would be expected based on molecular clock rates estimated from *Salmonella* epidemics or outbreaks 1, 52, 53, 54. The reason for the higher rate in the host is not known but may reflect either an innately higher mutation rate *in vivo* or reflect a higher replication rate in the host compared to in the environment during transmission between hosts. A large number of SNPs resulted in changes in coding sequence of proteins, including some involved in transcriptional regulation and virulence, that in some cases resulted in altered pathogenicity, consistent with adaptation 50, 54. Furthermore, a strain that persisted for several weeks in a patient contained ten SNPs in the *shdA* gene that encodes a surface localized fibronectin-binding protein involved in intestinal persistence 55, 56. Persistence for relatively short periods of time, 1–3 months, was also associated with HGT in the form of changes in prophage and plasmids 50, 57. In a well documented case of disseminated disease caused by *S*. Typhiumuirum ST313 in Malawi, a strain from a recrudescent infection, that had an identical genome sequence to the strain initially isolated, had acquired an IncHI2 plasmid conferring resistance to ceftriaxone [57]. Third-generation cephalosporins such as ceftriaxone had become a key antibiotic in treatment since the widespread emergence of the multidrug-resistant (MDR) *S*. Typhimurium ST313 epidemic clone. This was the first recorded case of an MDR *S*. Typhimurium ST313 strain expressing extended-spectrum β-lactamase (ESBL). The site of **recrudescence** in the patient was not known, although iNTS infection is generally disseminated, with *Salmonella* isolation from the blood and bone marrow being the most common [40]. The opportunity for variation arising from HGT would therefore appear to be limited, and ESBL-expressing *Salmonella* were exceedingly rare in the region. However, enteric bacteria expressing ESBL were relatively common, appearing soon after the first use of ceftriaxone for the general treatment of sepsis, some years previously [58]. It is therefore plausible that treatment of this patient with ceftriaxone partially decolonised the intestine, providing a niche colonised by an enteric bacterium containing the IncHI2 plasmid, that was transferred to the recrudescent *S*. Typhimurium strain. Persistent infections are more common in typhoid fever, but the whole-genome sequence of sequential isolates has not been determined to date. However, genomic rearrangements due to recombination between rRNA genes has been reported in sequential isolates from typhoid carriers, but was not observed during culture *in vitro*, suggesting a role in adaption to long-term carriage [59].

The potential of whole-genome sequencing to inform future clinical management of complicated infections, or even proactive management during an ongoing infection, is apparent from two well documented cases 60, 61. In the first, a patient with IL-12/23 β1 receptor deficiency was found to have a chronic persistent bacteraemia spanning over 15 years, due to a single infecting clone [61] (Box 3). The *S*. Enteritidis infection appeared to rapidly adapt to a systemic infection lifestyle in the immune compromised patient, resulting in increasingly occult bacteraemia. Despite the clonal nature of the infecting *S*. Enteritidis, sequential isolates over a 10-year period exhibited considerably greater genome sequence diversity than was expected, due to a **hypermutator** phenotype. Although recurrent infections with *Salmonella* are not uncommon in immunocompromised patients, they are normally resolved by antibiotic treatment. Yet in this case, repeated antimicrobial therapy resulted in the rapid emergence of resistance, suggesting that hypermutation was linked to the treatment failure, and suggested that alternative clinical management was appropriate. In a second documented case, the whole-genome sequence of a series of *S.* Typhimurium strains isolated over a 5-month period from a patient with a chronic infection for which antibiotic treatment ultimately failed, led to insight into the mechanism of treatment failure and contributed to the mechanistic understanding of drug resistance 62, 63. Importantly, pre- and post-therapy isolates were investigated, and the last isolate was found to contain a novel substitution in the AcrB protein, a component of a tripartite MDR efflux pump. The mutation altered the substrate specificity of the pump by affecting the drug-binding pocket, resulting in increased accumulation of ciprofloxacin, a key drug in the therapy of the patient, while decreasing accumulation of other drugs [60]. Evidence for how within-host evolution results in altered substrate specificities has the potential to inform effective treatment combinations in future complicated infections.

Box 3 **Within-Host Evolution during a Chronic Bacteraemia Infection**

In a rare clinical case, a patient with IL-12/23 β1 receptor deficiency presented with recurring bacteraemia spanning 15 years, despite repeated antibiotic and interferon(IF)-γ treatments [61] (Figure I). The whole-genome sequence revealed diversification with a molecular clock rate of 1 × 10^−5^ SNPs per site per year, around 50-fold higher than that observed in *Salmonella* epidemics over similar time periods 1, 52, 53. Rapid mutation was due to deletion within the *mutS* gene, conferring a hypermutator phenotype. All the patient isolates shared a set of mutations in common, consistent with early selection of mutations affecting phenotypes that conferred a strong advantage over progenitor variants. Later in infection, evolution resulted in multiple lineages defined by mutations present in one or a subset of isolates, likely to not confer such a robust advantage. Such early emergence of beneficial mutations and the rapid reduction in the relative advantage of subsequent mutations was also observed in experimental evolution of *E. coli* during adaptation *in vitro* [102]. Purifying selection dominated overall, as dN/dS <1, suggesting that many mutations had no selective advantage and were therefore lost from the population. However, dN/dS was greater than that observed for *S*. Enteritidis populations evolving with a normal lifestyle, suggesting that diversifying selection associated with adaptation was evident. A relatively low dN/dS is typical within evolving populations of *S*. Typhimurium, including during epidemics and outbreaks over evolutionary time scales, similar to that observed in the patient [1]. A notable exception is in *S*. Typhi populations, that, like the *S*. Enteritidis in the patient, have an elevated dN/dS, approaching unity [103], consistent with selection for some nonsynonymous mutations as they confer an advantage. That functional changes were under the influence of diversifying selection in the isolates from the patient was also suggested by the observation that proteins were more likely to contain substitutions in highly conserved functional domains, compared to *S*. Enteritidis evolving through gastroenteritis infections 61, 98. Many of these mutations are likely to be loss-of-function mutations in proteins that were no longer required for the infection, a characteristic of in-host adaptation of *Salmonella* serovars such as *S*. Typhi and *S*. Gallinarum 31, 98. Loss-of-function mutations were also evident in the presence of pseudogenes, similar in number and their functional classification to that reported for *S*. Typhi, and consistent with observed attenuation of virulence on challenge of subsequent hosts 14, 31.

Alt-text: Box 3

**Figure I:**
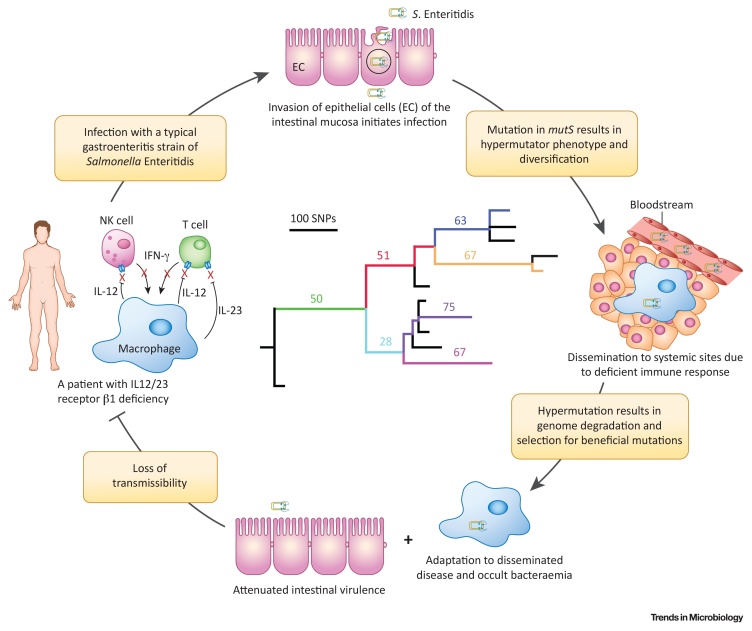
Within-Host Evolution of *S*. Enteritidis during Clinical Infection. Summary of disease progression and within-host evolution in a patient with interleukin-12/23 (IL 12/23) β1 receptor deficiency. A maximum likelihood tree based on core genome sequence variation of 11 sequential strains isolated from the blood of the patient over a 10-year period, and a typical *S*. Enteritidis PT4 strain (centre), as reported previously [61]. Numerals and colored edges in the phylogenetic tree indicate the number of pseudogenes that were introduced [61].

### Within-Host Evolution in Experimental Infections

The observation of within-host evolution in clinical infections is limited by the lack of routine whole-genome sequencing of clinical isolates, and the rarity of chronic infections. Clinical investigations are also, by necessity, restricted to observing evolution during the course of an infection within the confines of clinical management, and carefully controlled experimentation is not possible, complicating interpretation. An alternative is the employment of animal models of infection, a wide range of which are well characterized for *Salmonella* 49, 64, in which selection pressure can be finely controlled. In particular, the impact of HGT on transfer of antibiotic resistance, or virulence genes, has been studied using the murine infection model 33, 65.

## HGT within the Host

HGT is mediated by a number of mechanisms including conjugation, transformation, and phage-mediated transduction or **lysogeny** [66] (Box 2). The potential for HGT during intestinal infections is considerable due to the complex and numerous intestinal microbiota 67, 68. Conjugation is the dominant mechanism, used by ICEs and plasmids, but likely plays an important role in transfer of other mobile genetic elements (MGEs), such as transposons. Antibiotic-resistance genes are present on the chromosome or on plasmids in *Salmonella*, often associated with MGEs such as composite transposons or ICEs 14, 37, 69, 70, 71, 72. A number of factors impact the frequency of plasmid transfer. In *Escherichia coli*, conjugation was found to predominantly occur in the mucus layer, probably due to the stabilising properties of this matrix [73]. Proximity of donor and recipient is also an important factor for conjugation, and the density of cells plays an important role in the frequency of transfer [74]. Inflammation induced by *Salmonella* during colitis results in a proteobacterial bloom, including outgrowth of the pathogen 5, 75, thereby bringing related species into close proximity. These conditions enhanced the transfer of plasmid pII between *Salmonella* and commensal *E. coli* [33]. Many *Salmonella* serotypes encode an IncF plasmid called pSLT, or the virulence plasmid, due to the presence of *spv* genes required for systemic infections in mice. Transfer of pSLT occurs at low frequency during *in vitro* culture [76] but increases markedly in the mouse intestine, in response to high osmolarity and microaerobiosis 65, 77. Furthermore, transfer is considerably higher in the intestine compared to systemic sites of infection, highlighting the importance of the intestinal phase of infection for conjugative transfer. Transfer of plasmids between *Salmonella* strains can occur directly, but the microbiota may also play an important role as an intermediary. A large plasmid from *S*. Infantis, conferring the MDR phenotype, was transferred to *E. coli* and even to Gram-positive species, such as *Lactobacillus reuteri*, in experimental infections of mice. The plasmid was then further transferred to a second *Salmonella* serovar, *S*. Typhimurium, demonstrating a complex network of HGT in the intestine [78].

Stress imposed on *Salmonella* and the microbiota by antibiotics, bile, and host inflammation boosts HGT, likely an adaptation of MGEs to maximise their replication [79]. This characteristic of MGEs contributes significantly to the spread of genetically linked antibiotic-resistance genes and virulence genes. In addition to increasing the density of *Salmonella* and other proteobacteria in the intestine, inflammation induces the SOS stress response in *Salmonella* through the action of hypochlorite, reactive oxygen and reactive nitrogen species from transmigrated luminal granulocytes. The response has long been known to activate prophage, **temperate phage** commonly found inserted in the genome of bacteria [80], and more recently, ICEs [81]. Inflammation in the intestinal lumen is therefore likely to result in an increase in the transfer of DNA between the microbiota and pathogens by generalised transduction, although this has not been reported to date. However, prophage in the chromosome of *Salmonella* commonly encodes virulence factors, including superoxide dismutase SodCI, and a number of type III secretion system effector proteins [82]. One such phage, SopEΦ, encodes the T3SS effector protein, SopE, that boosts host cell invasion and inflammation 7, 83, 84, 85. Transfer of SopEΦ between the chromosome of two cocolonizing *S*. Typhimurium strains, by a process called lysogenic conversion, was considerably increased in the inflamed intestine [79]. Lysogenic conversion by SopEΦ has been associated with epidemic clones of *S*. Typhimurium in the past [86], and a current pandemic MDR clone of *S*. 4,[5],12:i:– (monophasic *S*. Typhimurium) has acquired the *sopE* gene on multiple occasions on a novel phage termed mtmVΦ [69]. Acquisition was accompanied by further clonal expansion, and within a 5-year period the proportion of strains encoding *sopE* increased from around 0% to 40% [69]. Lysogenic conversion by a temperate phage during clonal expansion of the MDR *S*. 4,[5],12:i:– clone highlights the potential for microevolution over very short periods of time that changes the nature and potentially the course of an epidemic.

## Concluding Remarks and Future Perspective

The revolution in recombinant DNA technology in the 20th century led to fruitful decades of research that employed molecular Koch’s postulates to study the molecular basis of bacterial pathogenicity [87]. With the advent of whole-genome sequencing technologies, determination of variation in whole-genome sequences that accompanies host adaptation of pathovariants, or within-host evolution under selection, has the potential to harness the naturally occurring variation and provides a complementary approach that not only provides insight into the mechanisms of pathogenesis, but also the emergence of new pathogens (see Outstanding Questions). Genome degradation associated with the evolution of host-adapted pathovars of *Salmonella* has revealed important metabolic activities specifically required for the intestinal phase of infection, but dispensable for disseminated disease. Direct observations of within-host evolution during chronic infections of individuals confirmed convergent pathways to host adaptation. Routine whole-genome sequencing of bacterial pathogens is being introduced in a number of countries for epidemiological surveillance. Its application in chronic and complicated *Salmonella* infections is a promising approach to provide data relevant to clinical management and insight into treatment failure.

Outstanding Questions Does *S*. Typhi evolve during chronic carriage in the gall bladder?

How does *S*. Typhi avoid loss of virulence and transmissibility during chronic carriage in the gall bladder?

What is the extent of HGT between coinfecting *Salmonella* and with the microbiota in the intestine during *Salmonella* gastroenteritis?

What are the barriers that limit intraspecies and intraphylum HGT in the intestine?

Does transient hypermutation contribute to the rapid emergence of new *Salmonella* pathovars?

## Glossary

**Disseminated disease**: infections that progress beyond the intestinal mucosa and associated lymphoid tissue.
**Diversifying selection**: change in frequency of a mutation due to its beneficial effect on the fitness of the organism.
**DT**: definitive type (phage type).
**Genetic drift**: changes in the frequency of mutations that are not under selection.
**Hypermutator**: a phenotype in which the normal substitution rate of an organism is elevated.
**Lysogeny**: the integration of bacteriophage DNA into the bacterial chromosome.
**Phage**: a virus particle that replicates within bacteria.
**Phage type**: a typing scheme based on the sensitivity of strains to lytic phage.
**Phase variation**: an heritable expression state of a protein frequently in an ON–OFF fashion.
**Purifying selection**: changes in the frequency of mutations due to their detrimental effect on the fitness of the organism.
**Recrudescence**: a recurring infection due to the same strain of pathogen that asymptomatically persists in the host.
**Serovar**: a variant of *Salmonella* based on intergenic variation of surface structures.
**Temperate phage**: a phage that is able to integrate into the bacterial genome and reside as a prophage.
**Typhoid fever**: disseminated disease caused by the host-restricted pathogen *Salmonella* Typhi.
